# Cross-Validation of a Scale to Measure Contact With Older Adults

**DOI:** 10.1093/geroni/igaa057.1038

**Published:** 2020-12-16

**Authors:** Robert Intrieri, Maria Kurth

**Affiliations:** 1 Western Illinois University, MACOMB, Illinois, United States; 2 Oregon State University, Corvallis, Oregon, United States

## Abstract

According to Allport (1954) intergroup contact would reduce prejudice that in-group members would experience toward out-group members. Allport also theorized that positive intergroup contact would follow after four conditions were met: (a) equal group status within the situation, (b) common goals, (c) intergroup cooperation, and (d) the support of authorities, law, or custom. While contact with older adults is a principal influence on attitudes toward older people, there is a paucity of adequate contact measures. This study assessed the cross-validity of the factor structure of an instrument to measure contact with older adults. The convenience sample consisted of 470 participants (61% male) from an undergraduate student subject pool (M = 20.67, SDtotal = 3.37). Participants were predominantly Caucasian (n=176, 48.9%), African American (n=103, 28.6%), and Hispanic/Latino/a (n=51, 14.2%). Results of a confirmatory factor analysis showed the three-factor model exhibited a reasonable fit to the data □□ (41, n = 360) = 191.797; p<.0001, CFI =.971; TLI =.961; RMSEA = .101 (90% CI, 0.087-0.116) SRMS = .042. An additional model examining the relationships between a single indicator of contact frequency and the three-factor COA scale revealed similar fit statistics □□ (41, n = 360) = 191.797; p<.0001, CFI =.967; TLI =.955; RMSEA = .092 (90% CI, 0.079-0.105) SRMS = .044. The findings provide clear and consistent evidence across independent samples that the covariances among the items are best explained through a latent structure that consists of three meaningful factors (“General Contact”, “Positive Experience, and “Negative Experience”).

